# Diagnostic accuracy of multiparametric MRI for detecting unconventional prostate cancer histology: a systematic review and meta-analysis

**DOI:** 10.1007/s00330-025-11603-3

**Published:** 2025-04-30

**Authors:** Filippo Carletti, Martina Maggi, Tamas Fazekas, Pawel Rajwa, Rossella Nicoletti, Jonathan Olivier, Felix Preisser, Timo F. W. Soeterik, Francesco Giganti, Alberto Martini, Isabel Heidegger, Veeru Kasivisvanathan, Benjamin Pradère, Guillaume Ploussard, Boris Hadaschik, Fabrizio Dal Moro, Roderick C. N. van den Bergh, Giancarlo Marra, Giorgio Gandaglia, Fabio Zattoni, Claudia Kesch

**Affiliations:** 1 Department of Surgery, Oncology and Gastroenterology, Urologic Unit, University of Padova, Padua, Italy; 2 Department of Maternal-Infant and Urological Sciences, Sapienza Rome University, Policlinico Umberto I Hospital, Rome, Italy; 3 Department of Urology, Comprehensive Cancer Center, Medical University of Vienna, Vienna, Austria; 4 Department of Urology, Semmelweis University, Budapest, Hungary; 5 Second Department of Urology, Centre of Postgraduate Medical Education, Warsaw, Poland; 6 Department of Experimental and Clinical Biomedical Science, University of Florence, Florence, Italy; 7 Department of Urology, CHU Lille, Lille, France; 8 Martini-Klinik Prostate Cancer Center, University Hospital Hamburg-Eppendorf, Hamburg, Germany; 9 Department of Radiation Oncology, University Medical Center, Utrecht, The Netherlands; 10 Department of Radiology, University College London Hospital NHS Foundation Trust, London, UK; 11 Division of Surgery and Interventional Science, University College London, London, England; 12 Department of Urology, University of Cincinnati, Cincinnati, US; 13 Department of Urology, Medical University Innsbruck, Innsbruck, Austria; 14 Department of Urology, La Croix Du Sud Hospital, Quint-Fonsegrives, France; 15 Department of Urology, University of Duisburg-Essen and German Cancer Consortium (DKTK)-University Hospital Essen, Essen, Germany; 16 Department of Urology, Erasmus University Medical Center, Rotterdam, The Netherlands; 17 Department of Urology, Città della Salute e della Scienza, University of Turin, Turin, Italy; 18 Unit of Urology/Division of Oncology, Gianfranco Soldera Prostate Cancer Lab, IRCCS San Raffaele Scientific Institute, Vita-Salute San Raffaele University, Milan, Italy; 19 Department of Medicine - DIMED, University of Padua, Padua, Italy

## Abstract

**Background and objective:**

Accurate detection of unconventional histologies (UH) in prostate cancer (PCa) is crucial for treatment planning and prognosis.

This systematic review and meta-analysis aimed to evaluate the accuracy of multiparametric magnetic resonance imaging (mpMRI) in detecting UH on prostatectomy, particularly cribriform architecture (CA) and intraductal carcinoma (IDC-P), in patients with localized PCa.

**Methods:**

A literature search was conducted in major databases for studies published after 2000. Seventeen articles fulfilled the inclusion criteria and were eligible for qualitative analysis. Five studies met the inclusion criteria for meta-analysis.

**Results:**

The pooled sensitivity and specificity of mpMRI (Prostate Imaging Reporting and Data System (PI-RADS) cutoff 3) to detect cribriform architecture were 0.91 and 0.29. The proportion of cribriform lesions increased with higher PI-RADS scores (23.2% for PI-RADS 1-2 to 66.7% for PI-RADS 5). For intraductal carcinoma (IDC-P), two studies found that IDC-P lesions were visible on mpMRI and had lower apparent diffusion coefficient (ADC) values compared to acinar prostate cancer. Four studies evaluating combined CA/IDC-P found sensitivities ranging from 33 to 100%. Lower ADC values were associated with CA/IDC-P in some studies, but not in others. Overall, mpMRI demonstrated promising sensitivity but moderate specificity in detecting these aggressive histological variants, with continued challenges in accurate sampling and characterization of mpMRI.

**Conclusions:**

mpMRI shows high sensitivity but moderate specificity in detecting cribriform architecture in PCa, especially for high PI-RADS scores. These findings support the use of mpMRI for UH detection, but caution is advised in clinical interpretation. Larger prospective studies are needed to validate these results before routine clinical application.

**Patient summary:**

We studied how effective MRI is at identifying different UH of PCa, such as cribriform architecture and intraductal carcinoma. MRI is accurate at detecting these cancers when they are present, but it also produces a significant number of false positives. More research is needed to standardize imaging protocols and histological definition and ensure an accurate diagnosis.

**Key Points:**

***Question*** *The accurate detection of unconventional histologies in prostate cancer, particularly cribriform architecture and intraductal carcinoma, is challenging but crucial for treatment planning and prognosis.*

***Findings*** *mpMRI shows high sensitivity (91%) but low specificity (29%) for detecting cribriform architecture, with detection rates increasing proportionally with higher PI-RADS scores.*

***Clinical relevance*** *mpMRI can effectively detect aggressive unconventional histologies in prostate cancer, though its moderate specificity suggests the need for careful interpretation. This aids in risk stratification and treatment planning, potentially improving patient outcomes.*

## Introduction

Prostate cancer (PCa) remains a significant global health challenge due to its heterogeneous nature, various histological subtypes with distinct biological and clinical behaviors [1, 2]. Among the available diagnostic tools, multiparametric magnetic resonance imaging (mpMRI) has emerged as the cornerstone for patients with suspected PCa, enhancing the detection of clinically significant PCa (csPCa) by means of targeted biopsies (TBx) [3]. The Prostate Imaging-Reporting and Data System (PI-RADS) classification [4] expresses the likelihood of csPCa using a 1-to-5 scale. However, its efficacy in identifying unconventional histologies (UH), such as cribriform architecture (CA), ductal, and intraductal prostate cancer (IDC-P) is unclear. UHs refer to specific growth patterns, histological types, and subtypes that differ from conventional acinar adenocarcinoma. The latest World Health Organization (WHO) classification indicates that while approximately 95% prostate cancer cases are diagnosed as conventional acinar adenocarcinoma, a significant minority (about 5%) exhibit UH [5], like cribriform architecture (CA), ductal, mucinous, PIN-like, squamous, sarcomatoid, neuroendocrine, signet-ring–like and intraductal prostate cancer (IDC-P). International Society of Urological Pathology (ISUP) defines CA as a Gleason score (GS) 4 pattern subtype that consists of a contiguous epithelial proliferation in which the majority of tumor cells do not contact adjacent stroma and display visible intercellular lumina [6]. There are two subtypes: small (also called round and simple) and large (also called expansile and confluent) cribriform glands [7], the definition and significance of which are still debated [8], but the large pattern seems to have the worst outcome [9]. Ductal adenocarcinoma is a highly aggressive histological subtype of PCa, which is composed of papillary structures and/or intricate cribriform glands lined by tall, columnar, pseudostratified epithelial cells. It most frequently occurs in the periurethral area [10]. Mucinous adenocarcinoma, a subtype of primary acinar adenocarcinoma, is characterized by the presence of extraluminal mucin comprising a minimum of 25% of the tumor volume [10]. PIN-like adenocarcinoma exhibits well-organized glands with short papillary infoldings lined by atypical epithelial cells [10], morphologically resembling high-grade prostatic intraepithelial neoplasia. The squamous group is composed of adenosquamous carcinoma, squamous cell carcinoma, and adenoid cystic (basal cell) carcinoma [10]. Sarcomatoid carcinoma is an exceedingly rare and highly aggressive variant, frequently arising in the context of high-grade adenocarcinoma, particularly following radiation therapy, and is associated with poor prognosis [10]. Neuroendocrine tumors are classified into well-differentiated neuroendocrine tumors, neuroendocrine carcinomas—including small cell, large cell, and mixed neuroendocrine neoplasms—and paraganglioma [10]. Signet-ring cell-like adenocarcinoma is characterized by intracytoplasmic vacuoles that displace the nucleus. This subtype is diagnosed when more than 25% of tumor cells exhibit signet-ring morphology and is rare, frequently associated with high Gleason patterns, and linked to poor clinical outcomes [10].

IDC-P is typically characterized by retrograde extension of high-grade cancer cells into preexisting non-neoplastic ducts and acini, distending them, with preservation of basal cells with solid, dense cribriform, loose cribriform and micropapillary architecture. There are controversies related to grading, nomenclature and the inclusion or exclusion in the Gleason grading system [10].

An accurate diagnosis of UH using mpMRI is crucial, as it directly influences staging, treatment planning, and patient outcomes, especially for aggressive UH like CA and IDC-P [2, 11–15]. The International Society of Urological Pathology (ISUP) [16] and the Genitourinary Pathology Society [17] have mandated the routine reporting of CA and IDC-P. Recent literature suggests that mpMRI parameters might require specific adjustments for precise UH characterization: the increased cellular density in CA/IDC-P may lead to restricted diffusion and low apparent diffusion coefficient (ADC) values, potentially reducing visibility on mpMRI [18–20]. Additionally, cribriform patterns tend to cluster densely within a single lesion rather than dispersing across multiple lesions [21]. However, whether CA and IDC-P present with atypical imaging features compared to more common adenocarcinomas remains poorly understood. Despite its clinical importance, there is currently no comprehensive evaluation in the literature synthesizing data on MRI performance across diverse UH. Therefore, our aim is to review the literature on the accuracy of mpMRI in detecting UH in localized PCa patients undergoing radical prostatectomy (RP).

## Evidence acquisition

### Protocol and methodology

The protocol has been registered in the International Prospective Register of Systematic Reviews database (registration ID: CRD42024521720). This systematic review was conducted following the Preferred Reporting Items for Systematic Reviews and Meta-analyses (PRISMA) statement (Supplementary Table 1).

### Study inclusion and exclusion criteria

Utilizing the Patient, Intervention, Comparison, Outcome (PICO) strategy, our investigation focused on PCa cases classified as cN0M0, exhibiting either mixed or purely UH. Our primary outcome was a per-lesion visibility of UH lesions on mpMRI. This review included prospective and retrospective studies that reported on patients diagnosed with CA, ductal, mucinous, PIN-like, squamous, sarcomatoid, neuroendocrine, signet-ring-like and IDC-P UH at prostate biopsy or RP, who received prior a mpMRI of the prostate. Eligible patients were those treated with RP for curative purposes. Studies involving neoadjuvant or adjuvant treatments were also considered. For cohorts reported multiple times, the dataset with the most comprehensive data was selected. Exclusion criteria were applied to studies that: (1) did not distinctly report mpMRI data for UH before definitive treatment of the primary tumor; (2) focused solely on prostate mpMRI before salvage treatments; (3) did not involve CA, ductal, mucinous, PIN-like, squamous, sarcomatoid, neuroendocrine, signet-ring-like and IDC-P UH; (4) used inappropriate pathological definitions for UH; (5) reported metastatic PCa; (6) did not utilize PI-RADS v2 or v2.1 for mpMRI interpretation.

### Search strategy and study selection

**Fig. 1:**
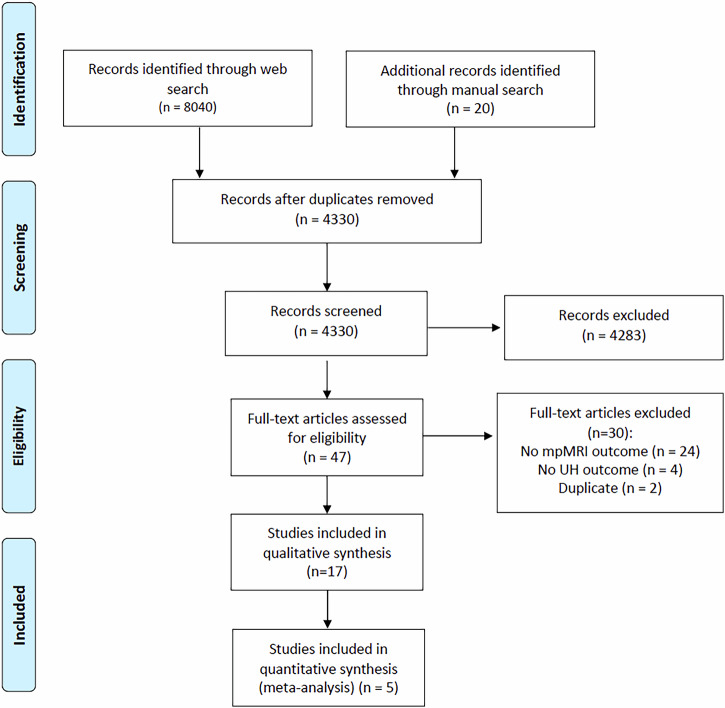
Preferred Reporting Items for Systematic Reviews and Meta-Analyses (PRISMA) flow diagram

The systematic review was performed according to the PRISMA guidelines (Fig. 1). On July 7, 2023, we searched PubMed/MEDLINE, Scopus and Web of Science Core Collection for records published since the year 2000. Additionally, we reviewed major urological journals and performed manual backwards citation searching to retrieve additional evidence, which resulted in the identification of 20 additional articles. The search strategy was performed using the following search string with free-text keywords and MeSH terms attached in Supplementary File 1. Four authors (C.K., F.Z., M.M., and F.C.) independently screened the records and extracted data, with any disagreements resolved by a third reviewer. The final data quality assessment was performed by two reviewers. Despite the reviewed studies being published prior to the 5th edition of the World Health Organization (WHO) classification, our findings are presented in line with this most recent edition. The term “Unconventional Histology” (UH) was collectively agreed upon to provide a generalized overview of our findings, as previously performed [2], even though it is not specifically mentioned in the WHO’s 5th edition, which delineates specific categories.

Extracted data consisted of study, population, and outcome characteristics, including publication year, country, sample size, MRI and biopsy protocol, basic patient characteristics, rates of (cs)PCa detection. Two by two diagnostic contingency tables were extracted or calculated from the studies containing true positive, false positive, true negative and false negative values, using mpMRI and pathologic evaluation as the index and reference tests for detecting CA in PCa lesions, respectively. Based on these counts, key diagnostic accuracy metrics were computed, including sensitivity and specificity. Studies were selected for qualitative analysis based on the inclusion criteria. Subsequently, those providing sufficient statistical data for sensitivity and specificity calculations were included in the quantitative analysis. This approach ensured that all relevant studies contributed to the overall synthesis while maintaining a rigorous standard for statistical pooling.

### Statistical analysis

Quantitative data synthesis was carried out with the packages “meta” and “metafor” of the R statistical software (R Core Team, 2019, R version 4.2.3), and the online tool described by Patel et al [22]. For our calculations, we followed the methods recommended by the working group of the Cochrane Collaboration [23]. The minimum number of studies to perform a meta-analysis was three, and for all analyses, a *p*-value of less than or equal to 0.05 was considered significant. Based on the likely heterogeneity of the studies, we utilized random-effect models for our calculations [24, 25]. To assess the proportion of cribriform histology of lesions in different PI-RADS categories, we calculated pooled event rates with 95% confidence intervals (CI) using the generalized mixed effect approach [26]. We used forest plots to visualize event rates and effect measures. Heterogeneity was assessed in the case of the pooled rates by calculating the I² measure and Cochran’s Q. To assess and plot summary estimates of sensitivity and specificity with 95% CI, we utilized bivariate random effects models and receiver operating characteristic curves (ROC), respectively [27, 28]. On the ROC curves, the sizes of the ellipsoids reflect the weights of the studies [29]. Publication bias could not be assessed due to the low number of articles (less than ten) for one outcome [30].

### Study quality assessment

Risk of bias in each study was assessed independently by the two investigators using the Quality assessment of Diagnostic Accuracy Studies-2 (QUADAS-2) tool [31]. The QUADAS-2 tool includes four domains—patient selection, index test, reference test, and time flow—which are all assessed in terms of risk of bias and the first three in terms of applicability. The questions used to score each domain were derived from the QUADAS-2 tool statement (Supplementary Table 2).

### Risk of bias using QUADAS-2

**Fig. 2:**
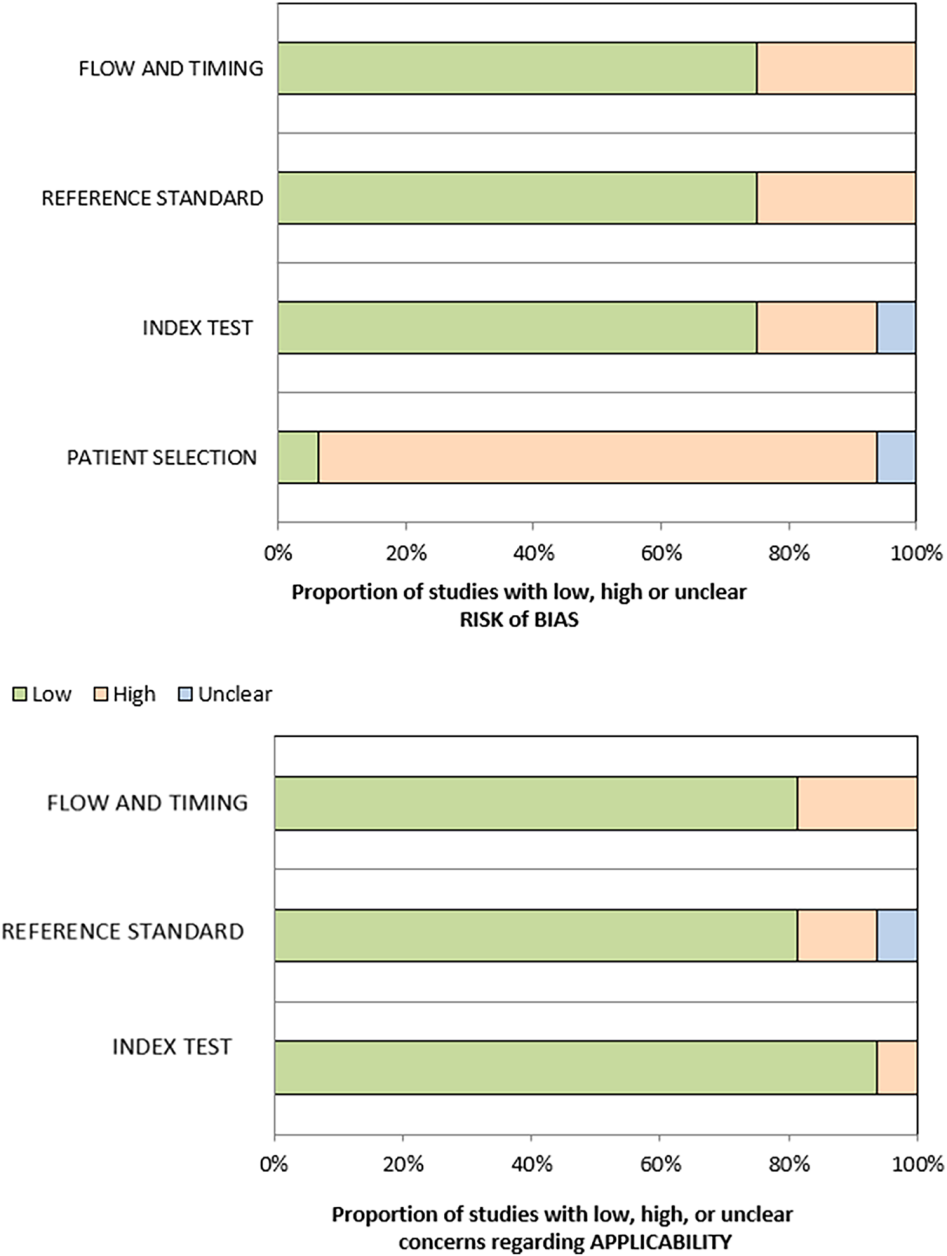
Summary of QUADAS-2 risk of bias assessments

The summary of RoB assessment and applicability concerns is presented in Fig. 2 and Supplementary Table 3. The applicability concerns of the studies were adequate in most cases.

Overall, the five studies included in this meta-analysis [12, 14, 17, 19, 24] had an intermediate risk of bias and low applicability concerns (Supplementary Table 3).

For Truong et al [32], high risk of bias and applicability concerns in the index domain were due to the ADC values obtained from the corresponding area of both visible and invisible cribriform lesions > 1 cm. Gao et al [33] high risk of selection bias was due to the inclusion of patients with Gleason pattern 4 with both MRI and [^68^Ga]Ga-PSMA PET/CT. Bilat et al [21] also had a high risk of bias in the patient selection due to the inclusion of a selected population of patients with cribriform architecture reported at final pathology. Cai et al’s [34] study had a high risk of bias and applicability concerns in the reference standard domain because it considered “cribriform morphology” as intraductal and/or Gleason grade 4 pattern with cribriform morphology. Arlsan et al [35] had a high risk of bias in the patient selection because of the population of patients with mpMRI with PI-RADS protocol, whole-mount PCa specimen and csPCa.

## Results

### Evidence synthesis

Seventeen articles fulfilled the inclusion criteria and were eligible for qualitative analysis. Of these [18, 21, 32–41], five were eligible for quantitative analysis [21, 32–35]. The majority of the studies were retrospective (*n* = 16), while 1 was prospective [42].

Five retrospective studies involving 279 patients with a total of 488 lesions (271 non-cribriform and 189 cribriform lesions) met the inclusion criteria for our meta-analysis [21, 32–35]. Notably, no studies were identified that included mucinous, PIN-like, adenosquamous, sarcomatoid, small cell neuroendocrine, or signet-ring–like subtypes. Table 1 provides a summary of the included studies in terms of baseline characteristics, methodology, and main findings with specific regard to CA pattern and MRI visibility. Of the five retrieved studies (Table 2), four reported CA and IDC-P in their analysis as recommended by the 2014 ISUP consensus conference [21, 32, 33, 35] and one [34] as recommended by the 2019 GUPS white paper. Images were acquired using 3-T MR scanners in all studies. PI-RADS v.2 was used for four studies [21, 32–34] while one study used PI-RADS version 2.1 [35]. PCa was confirmed by pathology using RP specimen.

**Table 1:** Baseline characteristics and methodology of the included studies (*n* = 17), reporting data on PCa UH and visibility at imaging

General study characteristics				Pathology						Imaging			
Author, reference, location	Study design	Years of accrual	Overall cohort: no. of pts	UH (definition)	No. of pathologists	Review	Blinded	Specimen for the diagnosis of UH	Bx technique	Imaging modality, PI-RADS version	Review	No. of radiologists	Blinded
Tonttila, [36], Finland	RETRO SC	2014–2016	124	CA and IDC-P (2014 ISUP consensus conference)	2	Yes	Yes	RP	-	3-T mpMRI, PI-RADS v2	Yes	2	Yes
Truong, [37], USA	RETRO SC	2014–2016	240	CA (2014 ISUP consensus conference)	1	Yes	Yes	Bx and RP	MRI/US Fusion TBx + 12 core SBx	3-T mpMRI, PI-RADS v2	No	2	No
Coffey, [38], Canada	RETRO SC	2012–2014	38	DA (*)	2	Yes	NR	RP	-	3-T mpMRI, PI-RADS v NR	Yes	2	Partly
Truong, [32], USA	RETRO SC	2015–2016	22	CA (2014 ISUP consensus conference)	1	Yes	Yes	Bx and RP	MRI/US Fusion TBx + 12-core SBx	3-T mpMRI, PI-RADS v2	No	2	No
Mannaerts, [39], USA	PROSP SC	2015–2018	142	CA and IDC-P (2014 ISUP consensus conference)	1	NR	NR	Bx	12-core SBx ± MRI/US Fusion TBx	1.5-T or 3-T mpMRI, PI-RADS v2	No	1	-
Currin, [40], Canada	RETRO SC	2013–2017	45	IDC -P (**)	1	Yes	NR	RP	-	3-T mpMRI, PI-RADS v2	Yes	2	Partly
Ericson, [52], USA	RETRO SC	2017–2018	455	CA and IDC-P (NR)	NR	Yes	Yes	Bx and RP	12-core SBx ± MRI/US Fusion TBx	mpMRI, PI-RADS v2	NR	NR	NR
Gao, [33], China	RETRO SC	2017–2018	49	CA (2014 ISUP consensus conference)	2	Yes	NR	RP	-	3-T mpMRI, PI-RADS v2	Yes	2	Yes
Hollemans, [44], The Netherlands	RETRO SC	2010–2017	51	Invasive CA and IDC-P (2014 ISUP consensus conference)	3	Yes	Yes	Bx and RP	SBx ± MRI/US Fusion TBx	mpMRI, PI-RADS v NR	NR	NR	NR
Prendeville, [18], Canada	RETRO SC	2013–2016	103	CA and IDC-P (2014 ISUP consensus conference)	2	Yes	NR	Bx	MRI/US Fusion TBx	3-T mpMRI, PI-RADS v2	Yes	2	Yes
Tuna, [21], Turkey	RETRO SC	2018–2021	33	CA and IDC-P (2014 ISUP consensus conference)	2	Yes	No	RP	-	3-T mpMRI, PI-RADS v2	Yes	2	Yes
Mikoshi, [43], Japan	RETRO SC	2007–2009	153	CA (Epstein JI. An update of the Gleason grading system. 2010) and IDC-P (McNeal’s criteria)	2	Yes	Yes	RP	-	1.5-T and 3-T mpMRI, PI-RADS v2.1	Yes	2	Yes
Cai, [34], USA	RETRO SC	2017–2019	117	CA and IDC-P (2019 GUPS white paper)	2	Yes	Yes	Bx and RP	In-bore Bx	3-T mpMRI, PI-RADS v2	No	1 out of 11	No
Pahouja, [45], USA	RETRO SC	2014–2021	281	DA or IDC-P	NR	NR	NR	Bx and RP	NR	NR	NR	NR	NR
Arslan, [35], Turkey	RETRO SC	2015–2020	58	CA and IDC -P (2014 ISUP consensus conference)	1	Yes	Yes	RP	-	3-T mpMRI, PI-RADS v2.1	Yes	2	Yes
Ghai, [42], Canada	PROSP SC	2019–2020	94	CA and IDC -P (NR)	2	Yes	NR	Bx	TBx (micro-US and MRI with FusionVu software) and SBx	3-T mpMRI, PI-RADS v2.1	No	1	-
Masoomian, [41], Canada	RETRO MC	2015–2018	69	CA (2014 ISUP consensus conference) and IDC-P (****)	6	No	NR	Bx and RP	NR	MRI	NR	NR	NR

*PCa* prostate cancer, *RETRO* retrospective, *SC* single center, *UH* unconventional histology, *CA* cribriform architecture, *IDC-P* intraductal carcinoma, *DA* ductal adenocarcinoma, *ISUP* International Society of Urological Pathology, *GUPS* Genitourinary Pathology Society, *Bx* biopsy, *RP* radical prostatectomy, *SBx* systematic Bx, *US* ultrasound, *mpMRI* multiparametric magnetic resonance imaging, *PI-RADS* Prostate Imaging-Reporting and Data System, *NR* not reported

* ≥ 50% invasive cells arrange in papillary projections or forming cribriform structures with slit-like lumen lined by malignant cells that are pseudostratified with abundant and typically amphophilic cytoplasm

** Prostatic adenocarcinoma spanning prostatic ducts and acini with intact or at least partially intact basal membranes on immunohistochemistry. The cells typically exhibit marked nuclear atypia and/or marked nucleomegaly (> 6 times normal)

*** Invasive cells arranged in papillary projections or forming cribriform structures with slit-like lumen. Malignant cells are pseudostratified and show abundant and typically amphophilic cytoplasm

**** Lumen spanning proliferation of carcinoma cells distending antecedent ducts or glands

**Table 2:** mpMRI visibility and mean ADC values of lesions with non-CA and CA at final pathology in the retrieved studies (*n* = 5)

	Non-CA lesions						CA lesions					
Author, reference, location	No.	GG at RP	Tumor size at pathology (mm):	PI-RADS score	VISIBLE at mpMRI, no. (%)	ADC mean	No.	GG at RP	Tumor size at pathology (mm):	PI-RADS score	VISIBLE at mpMRI, no. (%)	ADC mean
Truong, [32], USA	32	ISUP 1: 0 ISUP 2: 24 ISUP 3: 5 ISUP 4: 2 ISUP 5: 1	8*	1-2: 11 3: 6 4: 5 5: 10	21 (66)	773* µm^2^/s	18	ISUP 1: 0 ISUP 2: 7 ISUP 3: 9 ISUP 4: 2 ISUP 5: 0	12.5*	1-2: 13 3: 0 4: 1 5: 4	5 (28)	763* µm^2^/s
Gao, [33], China	25	ISUP 1: 0 ISUP 2: 20 ISUP 3: 4 ISUP 4: 1 ISUP 5: 0	14*	1-2: 3 3: 6 4: 13 5: 3	22 (88)	630§ µm^2^/s	37	ISUP 1: 0 ISUP 2: 11 ISUP 3: 23 ISUP 4: 3 ISUP 5: 0	18*	1-2: 1 3: 2 4: 21 5: 13	36 (97)	645§ µm^2^/s
Tuna, [21], Turkey	20	NR	NR	1-2: 0 3: 4 4: 12 5: 1	17 (85)	802* µm^2^/s	38	NR	NR	1-2: 0 3: 2 4: 19 5: 15	36 (95)	730* µm^2^/s
Cai, [34], USA	153	ISUP 1: 60 ISUP 2: 82 ISUP 3: 9 ISUP 4: 1 ISUP 5: 1	15*	1-2: 52 3: 12 4: 60 5: 29	101 (66)	0.609* mm/s*10^−3^	53	ISUP 1: 0 ISUP 2: 24 ISUP 3: 17 ISUP 4: 6 ISUP 5: 6	25*	1-2: 2 3: 1 4: 24 5: 26	51 (96)	0.557* mm/s*10^−3^
Arslan, [35], Turkey	41	NR	13.12§	1-2: 18 3-5: 37	37 (56)	0.688# mm^2^/s*10^−3^	43	ISUP 1: 0 ISUP 2: 11 ISUP 3: 14 ISUP 4: 4 ISUP 5: 14	12.35§	1-2: 17 3-5: 26	26 (60)	0.715# mm/ s*10^−3^
Overall data	271	-	-	-	198 (68)	-	189	-	-	-	154 (81)	-

*CA* cribriform architecture, *GG* ISUP grade group, *RP* radical prostatectomy, *mpMRI* multiparametric magnetic resonance imaging, *PI-RADS* Prostate Imaging-Reporting and Data System, *NR* not reported

* Median

# Unknown

§ Mean

### Visibility of cribriform pattern

**Fig. 3:**
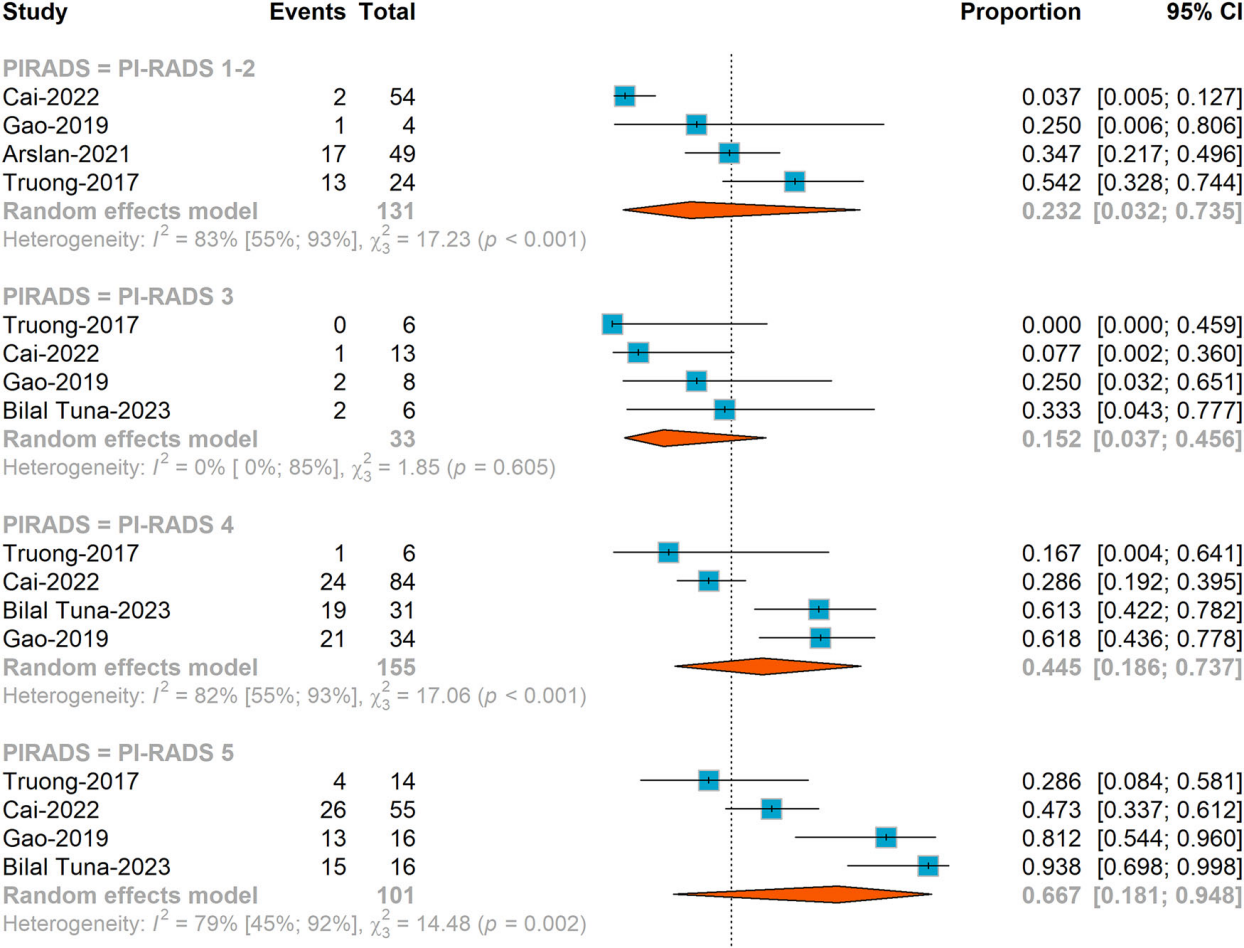
Proportion of lesions of cribriform adenocarcinoma in the different PI-RADS categories

While some studies suggest that mpMRI can be highly sensitive in detecting CA [21, 34], others indicate low visibility [32] or no significant differences [35]. Truong et al [37] demonstrated low visibility of pure cribriform pattern on mpMRI, with only 17.4% of tumors identified. Tuna et al [21] reported high sensitivity (94.7%) of mpMRI in detecting tumor foci with CA. The ADC values extracted from diffusion-weighted imaging (DWI) were significantly lower in CA areas compared to non-CA areas in single lesions. Cai et al [34] found high detection of CA by mpMRI, with 100% of index tumors and 78% of non-index tumors identified. However, in multivariate analyses, the presence of CA did not influence tumor visibility on mpMRI. Arslan et al [35] observed retrospectively that foci with CA component, foci without CA or IDC and foci with IDC-P had a similar invisibility rate on mpMRI (39.5%, 42.8%, and 21.4%, respectively; *p* = 0.11). Mikoshi et al [43] compared the histopathology between MRI-visible and MRI-invisible PCa among 153 patients (191 lesions) who had mpMRI and subsequent RP and found that there was no significant difference in the distribution of the CA (*p* > 0.99). We performed a quantitative synthesis on 5 studies [21, 32–35]. The prevalence of CA was 23.2% (95% CI: 3.2–73.5%), 15.2% (95% CI: 3.7–45.6%), 44.5% (95% CI: 18.6–73.7%) and 66.7% (95% CI: 18.1–94.8%) in PI-RADS 1-2, 3, 4, and 5 categories, respectively (Fig. 3). The pooled estimated sensitivity and specificity were 0.941 (95% CI: 0.456–0.997) and 0.233 (95% CI: 0.093–0.474) for PI-RADS 3-5, while 0.865 (95% CI: 0.545–0.971) and 0.425 (95% CI: 0.34–0.515) for PI-RADS 4-5, respectively (Table 3, Supplementary Figs. 1, 2).

**Table 3:** Pooled estimates of sensitivity and specificity for the detection of cribriform histology of PI-RADS 3-5 and 4-5

	PI-RADS 3-5	PI-RADS 4-5
Number of studies	5	4
Number of lesions	483	371
Sensitivity	0.941 (95% CI: 0.456–0.997)	0.865 (95% CI: 0.545–0.971)
Specificity	0.233 (95% CI: 0.093–0.474)	0.425 (95% CI: 0.34–0.515)

### Visibility of pure intraductal pattern

Two studies were included in the qualitative analysis, both showed that IDC-P lesions are visible and have PI-RADS ≥ 3 [35, 40].

Within a retrospective case control study involving 15 patients, Currin et al [40] reported that IDC-P, compared to acinar PCa, presents with lower ADC values in ISUP II and III tumors and higher PI-RADS score. Arslan et al [35] demonstrated that among 28 foci with an intraductal component, 6/28 (25%) were scored as PI-RADS 2, while 22/28 (75%) were scored as PI-RADS ≥ 3. IDC-P foci were larger than conventional foci, and there was no significant difference in the anatomical (i.e., peripheral vs central vs transitional, or base vs midgland, vs apex) location. Although IDC-P tumors exhibited lower ADC values than CA tumors, no significant difference was observed among groups with or without CA or IDC-P.

### Visibility of intraductal and cribriform pattern

Some studies have combined CA/IDC-P for different reasons. First, assessing mixed disease is methodologically easier and more clinically realistic instead of pure CA and pure IDC-P; second, it is often difficult to morphologically distinguish between CA and IDC-P and others without immunohistochemistry which is not systematically performed; third, the prognosis of the two is similar and comparable and their distinction seems less clinically relevant.

Combined CA/IDC-P was evaluated in four studies [18, 36, 43, 44]. Three demonstrated a very high sensitivity for CA/IDC-P, ranging from 90.5 [36] to 96% [18] to 100% [44], while one showed a sensitivity of 33% [43]. ADC values across two studies found no significant differences between CA/IDC-P and non-CA/IDC-P tumor foci [18, 36]. In one study, Mikoshi et al [43] found that lower ADC values were not linked to the histological presence of IDC-P but, interestingly, to the low ratio of cancer cells and a high ratio of lumen or stroma. Hollemans et al [44] did not consider ADC values. In terms of visibility and PI-RADS scores, Tonttila et al and Prendeville et al [18, 36] found that lesions with a PI-RADS score of 5 had a higher probability of being CA/IDC-P compared to lesions with lower PI-RADS scores. In contrast, Hollemans [44] found that the PI-RADS score was not a predictor for CA/IDC-P. In one study, this aspect was not discussed [43].

### Visibility of ductal adenocarcinoma

Two articles were considered for ductal adenocarcinoma [38, 45]. Coffey et al suggest that while ductal adenocarcinomas may be challenging to detect on T2-weighted imaging alone [38], they can be visible on dynamic contrast enhanced and often present with low ADC values on DWI [38]. Pahouja et al [45] reported that 90.7% of patients with ductal/IDC-P had a PI-RADS ≥ 4.

## Discussion

In this systematic review, we evaluated the ability of mpMRI in detecting UH in PCa, focusing on CA and IDC-P, as visible or non-visible lesions. Our study stands out for several reasons:

First, we found high variability in mpMRI visibility rates of CA, ranging from 28 [32] to 97% [33]. The pooled sensitivity of 0.9 suggests the high efficacy of mpMRI in detecting CA lesions. However, the specificity of 0.3 and the PPV of 0.6 imply moderate accuracy in correctly identifying true cribriform lesions among those detected, potentially resulting in a substantial number of FP cases. These false positives are specifically related to the identification of CA and IDC-P variants, rather than to the general presence or absence of prostate cancer.

There is a directly proportional association between CA detection and PI-RADS scores, with the highest proportion in PI-RADS 5, but even at the highest score (i.e., PI-RADS 5 lesions), about a third of CA lesions may still be missed. Cribriform cancers may be slightly under-detected at lower PI-RADS scores and potentially overrepresented at PI-RADS 5. Therefore, prostate Bx and prostate MRI-guided prostate biopsies are prone to undersampling at lower PI-RADS scores.

Second, the morphology of different histologies can influence how tumors appear on MRI. Specifically, the lower ADC values observed in CA/IDC-P may stem from the highly cellular epithelium characteristic of these cancers. The increased cellular density within these tumors likely leads to restricted diffusion, resulting in reduced visibility on MRI scans. Notably, ADC values are significantly lower in areas exhibiting CA compared to non-CA areas within the same lesion, suggesting that cribriform pattern cells are densely clustered rather than dispersed across multiple lesions [21]. However, it should be noted that these unconventional histologies often display overlapping histological features, complicating precise classification without the aid of immunohistochemistry. This highlights the need for more standardized histological definitions and grading systems.

In our systematic review, all studies used mpMRI. Currently, biparametric MRI (i.e., MRI without intravenous injection of contrast) is emerging as a potential tool for the initial PCa diagnostic [46–48]. Image quality should always be of the highest standard (e.g., using the Prostate Imaging Quality score) [49], both in the biparametric and multiparametric sequence.

Artificial intelligence (AI) has the potential to enhance the assist MRI interpretations for non-experienced readers [50] and machine learning algorithms might be trained to recognize subtle imaging features associated with UH, potentially reducing diagnostic errors and improving lesion detection rates [51]. To address the challenges in reporting unconventional histologies, standardized reporting protocols incorporating AI tools could be developed. These protocols should integrate imaging data with pathological findings to provide a comprehensive diagnostic overview.

Selection bias and heterogeneity of outcomes significantly limit the generalizability of our systematic review. The included studies often employed highly specific inclusion criteria, and it does not include any mucinous, PIN-like, adenosquamous, sarcomatoid, small cell neuroendocrine, or signet-ring–like subtypes. For instance, Gao et al [33] only included patients who had undergone both MRI and PET scans, focusing on lesions with Gleason pattern 4 but not 5. Similarly, Tuna et al [21] specifically selected 33 patients with cribriform foci, which may not represent the broader PCa population.

A reporting bias should also be considered. Differences in MRI acquisition techniques, variations in contrast agent use and the level of expertise of the radiologists interpreting the images may contribute to the heterogeneity observed across studies. Additionally, not all pathologists are experts in identifying uncommon histological patterns, particularly for unconventional histologies (UH), and inconsistencies in histopathological reference standards between institutions may also influence the variability in reported diagnostic performance. This variability in expertise may lead to underreporting or overreporting of these features, potentially influencing the reported prevalence or incidence of conditions like IDC-P or CA.

Furthermore, our analysis is limited to patients who underwent RP, excluding those managed with other treatments. Consequently, our findings may not accurately represent the broader demographic of men with PCa.

The retrospective nature of the included studies compounds this bias, as they rely on existing data and may not capture a representative sample of the target population. Additionally, confounding factors such as the association between ISUP grade at RP and the presence of CA further complicate interpretation. These biases and confounding factors may significantly impact the precision and generalizability of our results. While we cannot fully remove these limitations given the nature of the available studies, it is essential to interpret our findings with caution. Future prospective studies with more diverse patient populations and standardized inclusion criteria are needed to more accurately assess the relationship between MRI findings and unconventional histologies of PCa.

## Conclusions

While mpMRI demonstrates promising sensitivity for detecting CA in PCa lesions, its moderate specificity and PPV suggest room for improvement. The correlation between PI-RADS scores and the likelihood of CA needs further investigation. Challenges remain in standardizing imaging protocols and histological classifications. Future research should prioritize studies with larger sample sizes and adopt prospective study designs to overcome the current limitations and refine the PI-RADS classification system.

## Supplementary information

ELECTRONIC SUPPLEMENTARY MATERIAL

## Abbreviations

ADC: Apparent diffusion coefficient
CA: Cribriform architecture
CI: Confidence intervals
csPCa: Clinically significant prostate cancer
IDC-P: Intraductal prostate cancer
ISUP: International Society of Urological Pathology
mpMRI: Multiparametric magnetic resonance imaging
PCa: Prostate cancer
RP: Radical prostatectomy
UH: Unconventional histologies

## References

[1] Sung H, Ferlay J, Siegel RL et al (2021) Global cancer statistics 2020: GLOBOCAN estimates of incidence and mortality worldwide for 36 cancers in 185 countries. CA Cancer J Clin 71:209–249. 10.3322/CAAC.21660 33538338 10.3322/caac.21660

[2] Marra G, van Leenders GJLH, Zattoni F et al (2023) Impact of epithelial histological types, subtypes, and growth patterns on oncological outcomes for patients with nonmetastatic prostate cancer treated with curative intent: a systematic review. Eur Urol 84:65–85. 10.1016/J.EURURO.2023.03.014 37117107 10.1016/j.eururo.2023.03.014

[3] Kasivisvanathan V, Rannikko AS, Borghi M et al (2018) MRI-targeted or standard biopsy for prostate-cancer diagnosis. N Engl J Med 378:1767–1777. 10.1056/NEJMOA1801993/SUPPL_FILE/NEJMOA1801993_DISCLOSURES.PDF 29552975 10.1056/NEJMoa1801993 PMC9084630

[4] Weinreb JC, Barentsz JO, Choyke PL et al (2016) PI-RADS Prostate Imaging-Reporting and Data System: 2015, version 2. Eur Urol 69:16–40. 10.1016/J.EURURO.2015.08.052 26427566 10.1016/j.eururo.2015.08.052 PMC6467207

[5] Netto GJ, Amin MB, Berney DM et al (2022) The 2022 World Health Organization classification of tumors of the urinary system and male genital organs—Part B: Prostate and urinary tract tumors. Eur Urol 82:469–482. 10.1016/J.EURURO.2022.07.002 35965208 10.1016/j.eururo.2022.07.002

[6] van der Kwast TH, van Leenders GJ, Berney DM et al (2021) ISUP consensus definition of cribriform pattern prostate cancer. Am J Surg Pathol 45:1118–1126. 10.1097/PAS.0000000000001728 33999555 10.1097/PAS.0000000000001728

[7] Trudel D, Downes MR, Sykes J, Kron KJ, Trachtenberg J, Van Der Kwast TH (2014) Prognostic impact of intraductal carcinoma and large cribriform carcinoma architecture after prostatectomy in a contemporary cohort. Eur J Cancer 50:1610–1616. 10.1016/j.ejca.2014.03.009 24703897 10.1016/j.ejca.2014.03.009

[8] Rijstenberg LL, Hansum T, Kweldam CF et al (2022) Large and small cribriform architecture have similar adverse clinical outcome on prostate cancer biopsies. Histopathology 80:1041. 10.1111/HIS.14658 35384019 10.1111/his.14658 PMC9321809

[9] Hollemans E, Verhoef EI, Bangma CH et al (2019) Large cribriform growth pattern identifies ISUP grade 2 prostate cancer at high risk for recurrence and metastasis. Mod Pathol 32:139–146. 10.1038/s41379-018-0157-9 30349027 10.1038/s41379-018-0157-9 PMC6300553

[10] Amin MB (2022) Urinary and male genital tumours. WHO Classification of Tumours Editorial Board, Lyon

[11] Kweldam CF, Wildhagen MF, Steyerberg EW, Bangma CH, Van Der Kwast TH, Van Leenders GJLH (2015) Cribriform growth is highly predictive for postoperative metastasis and disease-specific death in Gleason score 7 prostate cancer. Mod Pathol 28:457–464. 10.1038/modpathol.2014.116 25189638 10.1038/modpathol.2014.116

[12] Keefe DT, Schieda N, El Hallani S et al (2015) Cribriform morphology predicts upstaging after radical prostatectomy in patients with Gleason score 3 + 4 = 7 prostate cancer at transrectal ultrasound (TRUS)-guided needle biopsy. Virchows Arch 467:437–442. 10.1007/s00428-015-1809-5 26229020 10.1007/s00428-015-1809-5

[13] McNeal JE, Yemoto CEM (1996) Spread of adenocarcinoma within prostatic ducts and acini. Morphologic and clinical correlations. Am J Surg Pathol 20:802–814. 10.1097/00000478-199607000-00003 8669528 10.1097/00000478-199607000-00003

[14] Wilcox G, Soh S, Chakraborty S, Scardino PT, Wheeler TM (1998) Patterns of high-grade prostatic intraepithelial neoplasia associated with clinically aggressive prostate cancer. Hum Pathol 29:1119–1123. 10.1016/S0046-8177(98)90423-3 9781651 10.1016/s0046-8177(98)90423-3

[15] Cohen RJ, Chan WC, Edgar SG et al (1998) Prediction of pathological stage and clinical outcome in prostate cancer: an improved pre-operative model incorporating biopsy-determined intraductal carcinoma. Br J Urol 81:413–418. 10.1046/J.1464-410X.1998.00530.X 9523662 10.1046/j.1464-410x.1998.00530.x

[16] Iczkowski KA, Van Leenders GJLH, Van Der Kwast TH (2021) The 2019 International Society of Urological Pathology (ISUP) consensus conference on grading of prostatic carcinoma. Am J Surg Pathol 45:1005–1007. 10.1097/PAS.0000000000001678 33481387 10.1097/PAS.0000000000001678

[17] Epstein JI, Amin MB, Fine SW et al (2021) The 2019 genitourinary pathology society (GUPS) white paper on contemporary grading of prostate cancer. Arch Pathol Lab Med 145:461–493. 10.5858/arpa.2020-0015-ra 32589068 10.5858/arpa.2020-0015-RA

[18] Prendeville S, Gertner M, Maganti M et al (2018) Role of magnetic resonance imaging targeted biopsy in detection of prostate cancer harboring adverse pathological features of intraductal carcinoma and invasive cribriform carcinoma. J Urol 200:104–113. 10.1016/j.juro.2018.01.081 29408568 10.1016/j.juro.2018.01.081

[19] Langer DL, van der Kwast TH, Evans AJ et al (2010) Prostate tissue composition and MR measurements: investigating the relationships between ADC, T2, K(trans), v(e), and corresponding histologic features. Radiology 255:485–494. 10.1148/RADIOL.10091343 20413761 10.1148/radiol.10091343

[20] Helfrich O, Puech P, Betrouni N et al (2017) Quantified analysis of histological components and architectural patterns of Gleason grades in apparent diffusion coefficient restricted areas upon diffusion weighted MRI for peripheral or transition zone cancer locations. J Magn Reson Imaging 46:1786–1796. 10.1002/JMRI.25716 28383776 10.1002/jmri.25716

[21] Tuna MB, Arslan A, Kök YB et al (2023) Cribriform pattern of the prostate adenocarcinoma: sensitivity of multiparametric MRI. Urol J 20:34–40. 10.22037/uj.v19i.7382 10.22037/uj.v19i.7382 36528799

[22] Patel A, Cooper N, Freeman S, Sutton A (2021) Graphical enhancements to summary receiver operating characteristic plots to facilitate the analysis and reporting of meta-analysis of diagnostic test accuracy data. Res Synth Methods 12:34–44. 10.1002/JRSM.1439 32706182 10.1002/jrsm.1439

[23] Green S, Higgins JPT, Alderson P, Clarke M, Mulrow CD, Oxman AD (2008) Chapter1: Introduction. In: Higgins JPT, Green S (eds) Cochrane handbook for systematic reviews of interventions. John Wiley & Sons, Chichester

[24] Assel M, Sjoberg D, Elders A et al (2019) Guidelines for reporting of statistics for clinical research in urology. BJU Int 123:401–410. 10.1111/BJU.14640 30537407 10.1111/bju.14640 PMC6397060

[25] Tufanaru C, Munn Z, Stephenson M, Aromataris E (2015) Fixed or random effects meta-analysis? Common methodological issues in systematic reviews of effectiveness. Int J Evid Based Healthc 13:196–207. 10.1097/XEB.0000000000000065 26355603 10.1097/XEB.0000000000000065

[26] Stijnen T, Hamza TH, Özdemir P (2010) Random effects meta-analysis of event outcome in the framework of the generalized linear mixed model with applications in sparse data. Stat Med 29:3046–3067. 10.1002/SIM.4040 20827667 10.1002/sim.4040

[27] Reitsma JB, Glas AS, Rutjes AWS, Scholten RJPM, Bossuyt PM, Zwinderman AH (2005) Bivariate analysis of sensitivity and specificity produces informative summary measures in diagnostic reviews. J Clin Epidemiol 58:982–990. 10.1016/J.JCLINEPI.2005.02.022 16168343 10.1016/j.jclinepi.2005.02.022

[28] Chu H, Cole SR (2006) Bivariate meta-analysis of sensitivity and specificity with sparse data: a generalized linear mixed model approach. J Clin Epidemiol 59:1331–1332. 10.1016/j.jclinepi.2006.06.011 17098577 10.1016/j.jclinepi.2006.06.011

[29] Burke DL, Ensor J, Snell KIE, van der Windt D, Riley RD (2018) Guidance for deriving and presenting percentage study weights in meta-analysis of test accuracy studies. Res Synth Methods 9:163–178. 10.1002/JRSM.1283 29115060 10.1002/jrsm.1283

[30] Sterne JA, Sutton AJ, Ioannidis JP et al (2011) Recommendations for examining and interpreting funnel plot asymmetry in meta-analyses of randomised controlled trials. BMJ. 10.1136/BMJ.D4002 10.1136/bmj.d4002 21784880

[31] Whiting PF, Rutjes AW, Westwood ME et al (2011) QUADAS-2: a revised tool for the quality assessment of diagnostic accuracy studies. Ann Intern Med 155:529–536. 10.7326/0003-4819-155-8-201110180-00009 22007046 10.7326/0003-4819-155-8-201110180-00009

[32] Truong M, Hollenberg G, Weinberg E, Messing EM, Miyamoto H, Frye TP (2017) Impact of Gleason subtype on prostate cancer detection using multiparametric magnetic resonance imaging: correlation with final histopathology. J Urol 198:316–321. 10.1016/j.juro.2017.01.077 28163032 10.1016/j.juro.2017.01.077

[33] Gao J, Zhang C, Zhang Q et al (2019) Diagnostic performance of ^68^Ga-PSMA PET/CT for identification of aggressive cribriform morphology in prostate cancer with whole-mount sections. Eur J Nucl Med Mol Imaging 46:1531–1541. 10.1007/s00259-019-04320-9 31025048 10.1007/s00259-019-04320-9

[34] Cai Q, Costa DN, Metter CK et al (2022) Sensitivity of multiparametric MRI and targeted biopsy for detection of adverse pathologies (cribriform Gleason pattern 4 and intraductal carcinoma): correlation of detected and missed prostate cancer foci with whole mount histopathology. Urol Oncol 40:452.e1–452.e8. 10.1016/j.urolonc.2022.07.012 36008255 10.1016/j.urolonc.2022.07.012

[35] Arslan A, Alis D, Tuna MB, Sağlıcan Y, Kural AR, Karaarslan E (2021) The visibility of prostate cancer concerning underlying histopathological variances: a single-center multiparametric magnetic resonance imaging study. Eur J Radiol. 10.1016/j.ejrad.2021.109791 10.1016/j.ejrad.2021.109791 34062471

[36] Tonttila PP, Ahtikoski A, Kuisma M, Pääkkö E, Hirvikoski P, Vaarala MH (2019) Multiparametric MRI prior to radical prostatectomy identifies intraductal and cribriform growth patterns in prostate cancer. BJU Int 124:992–998. 10.1111/bju.14812 31102571 10.1111/bju.14812

[37] Truong M, Feng C, Hollenberg G et al (2018) A comprehensive analysis of cribriform morphology on magnetic resonance imaging/ultrasound fusion biopsy correlated with radical prostatectomy specimens. J Urol 199:106–113. 10.1016/j.juro.2017.07.037 28728994 10.1016/j.juro.2017.07.037

[38] Coffey N, Schieda N, Cron G, Gulavita P, Mai KT, Flood TA (2015) Multi-parametric (mp) MRI of prostatic ductal adenocarcinoma. J Magn Reson Imaging 41:1639–1645. 10.1002/jmri.24694 25044687 10.1002/jmri.24694

[39] Mannaerts CK, Engelbrecht MRW, Postema AW et al (2020) Detection of clinically significant prostate cancer in biopsy-naïve men: direct comparison of systematic biopsy, multiparametric MRI- and contrast-ultrasound-dispersion imaging-targeted biopsy. BJU Int 126:481–493. 10.1111/bju.15093 32315112 10.1111/bju.15093

[40] Currin S, Flood TA, Krishna S, Ansari A, McInnes MDF, Schieda N (2019) Intraductal carcinoma of the prostate (IDC-P) lowers apparent diffusion coefficient (ADC) values among intermediate risk prostate cancers. J Magn Reson Imaging 50:279–287. 10.1002/jmri.26594 30585372 10.1002/jmri.26594

[41] Masoomian M, Downes MR, Sweet J et al (2019) Concordance of biopsy and prostatectomy diagnosis of intraductal and cribriform carcinoma in a prospectively collected data set. Histopathology 74:474–482. 10.1111/his.13747 30160779 10.1111/his.13747

[42] Ghai S, Perlis N, Atallah C et al (2022) Comparison of micro-US and multiparametric MRI for prostate cancer detection in biopsy-naive men. Radiology 305:390–398. 10.1148/radiol.212163 35852425 10.1148/radiol.212163

[43] Mikoshi A, Miyai K, Hamabe F et al (2022) MRI-detectability and histological factors of prostate cancer including intraductal carcinoma and cribriform pattern. Prostate 82:452–463. 10.1002/pros.24291 34964158 10.1002/pros.24291

[44] Hollemans E, Verhoef EI, Bangma CH et al (2019) Concordance of cribriform architecture in matched prostate cancer biopsy and radical prostatectomy specimens. Histopathology 75:338–345. 10.1111/his.13893 31045262 10.1111/his.13893 PMC6851781

[45] Pahouja G, Patel HD, Desai S et al (2023) The rising incidence of ductal adenocarcinoma and intraductal carcinoma of the prostate: diagnostic accuracy of biopsy, MRI-visibility, and outcomes. Urol Oncol 41:48.e11–48.e18. 10.1016/j.urolonc.2022.09.025 36441068 10.1016/j.urolonc.2022.09.025

[46] Ng A, Khetrapal P, Kasivisvanathan V (2022) Is it PRIME time for biparametric magnetic resonance imaging in prostate cancer diagnosis? Eur Urol 82:1–2. 10.1016/j.eururo.2022.02.021 35277288 10.1016/j.eururo.2022.02.021

[47] Asif A, Nathan A, Ng A et al (2023) Comparing biparametric to multiparametric MRI in the diagnosis of clinically significant prostate cancer in biopsy-naive men (PRIME): a prospective, international, multicentre, non-inferiority within-patient, diagnostic yield trial protocol. BMJ Open 13:e070280. 10.1136/bmjopen-2022-070280 37019486 10.1136/bmjopen-2022-070280 PMC10083803

[48] Porter KK, King A, Galgano SJ, Sherrer RL, Gordetsky JB, Rais-Bahrami S (2020) Financial implications of biparametric prostate MRI. Prostate Cancer Prostatic Dis 23:88–93. 10.1038/s41391-019-0158-x 31239513 10.1038/s41391-019-0158-x

[49] de Rooij M, Allen C, Twilt JJ et al (2024) PI-QUAL version 2: an update of a standardised scoring system for the assessment of image quality of prostate MRI. Eur Radiol. 10.1007/s00330-024-10795-4 10.1007/s00330-024-10795-4 PMC11519155 38787428

[50] Saha A, Bosma JS, Twilt JJ et al (2024) Artificial intelligence and radiologists in prostate cancer detection on MRI (PI-CAI): an international, paired, non-inferiority, confirmatory study. Lancet Oncol 25:879–887. 10.1016/S1470-2045(24)00220-1 38876123 10.1016/S1470-2045(24)00220-1 PMC11587881

[51] Lenfant L, Seisen T, Rouprêt M, Pinar U, Mozer PC (2023) Unleashing the power of artificial intelligence and fusion magnetic resonance imaging-targeted biopsy: transforming prostate cancer diagnosis. Eur Urol Oncol 6:541–542. 10.1016/j.euo.2023.06.013 37586959 10.1016/j.euo.2023.06.013

[52] Ericson KJ, Wu SS, Lundy SD, Thomas LJ, Klein EA, McKenney JK (2020) Diagnostic accuracy of prostate biopsy for detecting cribriform Gleason pattern 4 carcinoma and intraductal carcinoma in paired radical prostatectomy specimens: implications for active surveillance. J Urol 203:311–317. 10.1097/JU.0000000000000526 31483693 10.1097/JU.0000000000000526

